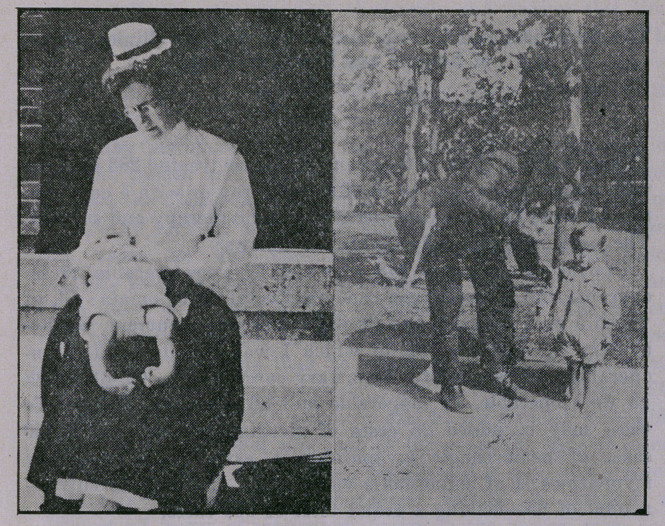# The Treatment of Congenital Club-foot

**Published:** 1919-04

**Authors:** Willis C. Campbell

**Affiliations:** 1028 Exchange Building, Memphis. Tenn.


### CONTRIBUTED ARTICLES.

### The Treatment of Congenital Club-Foot.

WILLIS C. CAMPBELL, M. D., F. A. C. S.,
1028 Exchange Building, Memphis. Tenn.

Congenital club-foot, or equinus-varus, is a common anomaly, the treatment of which is often inefficient and inadequate, though very easily corrected if the mechanical principles are thoroughly understood and properly applied for sufficient time to prevent recurrence. A foot may be apparently corrected to the untrained eye by simply severing the tendo Achillis, but unless due consideration is given to the distorted bony contour, as well as muscle balance, recurrence is inevitable.

The routine treatment differs materially as to age, degree and character. At birth simple adhesive may ease the mind of the mother but not until two weeks have elapsed is it convenient to begin active treatment, which consists in gradual correction of first the varus or in toe, and later the equinus or drop foot. For this successive plaster casts are used. Great care must be exercised not to rush correction, as sloughing may occur or serious interference with the blood supply. Casts extend from toes to knee except in fat babies where the knee at right ankle is included. Each cast remains for one week, on removal of which massage and gentle manipulations are given. From four to six over correct the varus and about four the equinus. Care is also taken to correct cavus or high arch and rotation of the calcaneum at the calcaneo-astragaloid joint. When full correction is attained casts are changed at the end of one month and a retentive brace applied, provided the mother can be trusted to carry out accurately passive moments, otherwise the cast is continued at monthly intervals until permanent over-correction is attained. As soon as standing is acquired shoes are raised one-eighth to one-fourth an inch on the outer front aspect, and the braces worn during rest periods and at night. Walking is encouraged as early as possible, but the passive movements are continued for one or more years.

In those above one year operative procedures are indicated though excellent results can be attained by gradual correction

This and illustrations that follow show the results of treatment as explained by Dr. Campbell. The ages of patients varied from infancy to old age and results were obtained in from six months to a year.

in many much older, however, the length of time required prohibits the usual neglected or relapsed case from such treatment. For the average one to five years and in some younger than one year, forcible correction is done by hand or over wedge shaped block, over correcting first the varus and then equinus by plastic lengthening of the tendo Achillis. Except in the very young ordinary subcutaneous tenotomy should not be practiced as dense scar tissue may impede the action of the muscle or cause relapse of equinus. In those of severe degree perfect correction cannot always be accomplished by this method, and the removal of redundant bone in the tarsus may later be necessary, however, the amount of such bone to be removed can be appreciably reduced by forcible correction and a practically normal foot attained.

In old and relapsed cases open operative procedures are necessary. In fact, no age exists in which excellent correction cannot be secured. As a general rule, however, the older the individual the less resilient and elastic foot, but a perfectly straight foot which will fit the ordinary shoe can be definitely promised. The operation which I have done for years is as follows:

An incision is made over the most prominent portion of the tarsus from above downward, the usual large bursal sack is disected out intact, as many of these are infected. The deep fascia and periosteum is next incised and a wedge removed, consisting of the head of the astragalus in all cases, and often a portion of the anterior extremity of the os calcis, and posterior aspects of the cuboid and scaphoid. The varus is now easily corrected or sufficient bone removed until desired position is attained. So far the method is the old tarsectomy recently advocated by Cook of Hartford. The next step I have devised and consider of material advantage in correction of the heel and relation of the foot to the leg. The posterior wall of the incision is formed by the anterior raw surfaces of the astragalus above and calcaneum below, with articulations and ligaments intervening. The chisel now passes directly backward remodeling the interior surface of the astragalus and superior surface of the os calcis, removing the articulation and intervening ligaments when the entire foot is abducted and dorsi-flexed. If the external maleolus is posteriorly placed the knife severs the external ligaments between this bone and the tarsus. Over-correction is now attained unless equinus is excessive when tenoplasty of the Achilles completes the operation. A retentive brace or plaster cast is worn for eight weeks after which no further treatment except observation is needed.

There are many advocates of other methods and undoubtedly complete correction can be made in old and severe deformation by successive forcible manipulations or gradual force by apparatus, but such treatment requires more time, pain, suffering and expense than the average patient is either willing or able to give. A number of surgeons are radical in the treatment of all cases submitting even very young children to open bone operations. It has been my practice to pursue a middle course for we must consider conditions present in the individual case, such as character, intelligence and financial ability of the parents or patient to pursue a long continued treatment. We should always be as conservative as possible, and avoid the removal of bone, for the less we disturb the small joints of the tarsus the nearer normal the foot, the more elastic the step. All cases should be treated if possible from birth, when a perfect result can be invariably obtained with cooperation of the parents. No case should be allowed to go through life with this hideous deformity, as a perfectly stable, useful straight foot will avoid much physical suffering and inflammatory bursa and periostitis to say nothing of the constant mental embarrassment.

No case reports are given, as accompanying exhibits of each type are sufficient.

## Figures and Tables

**Figure f1:**
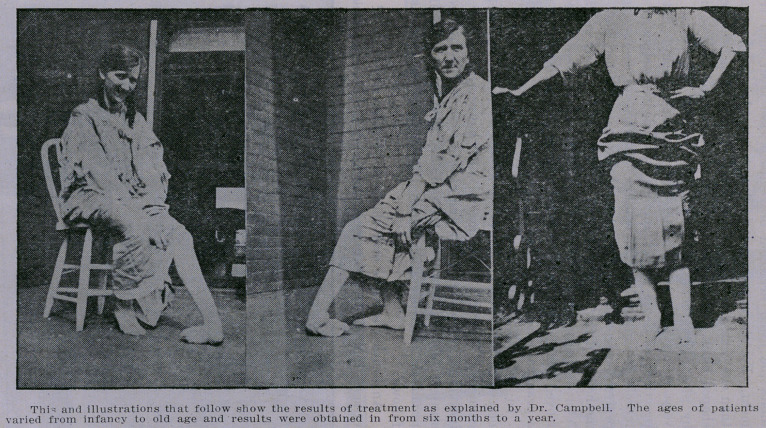


**Figure f2:**
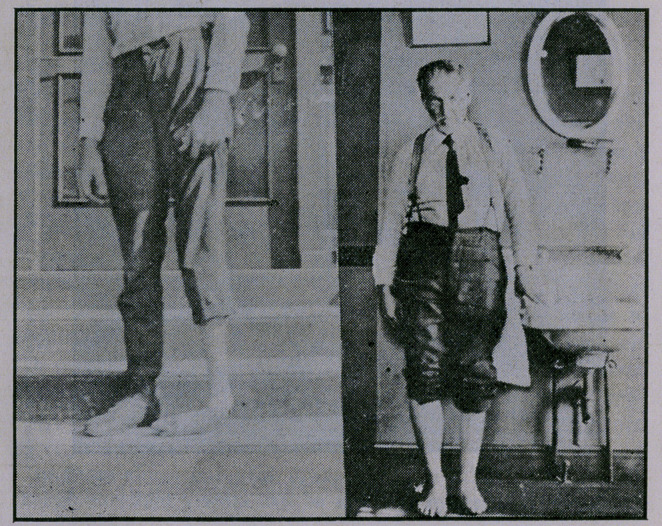


**Figure f3:**
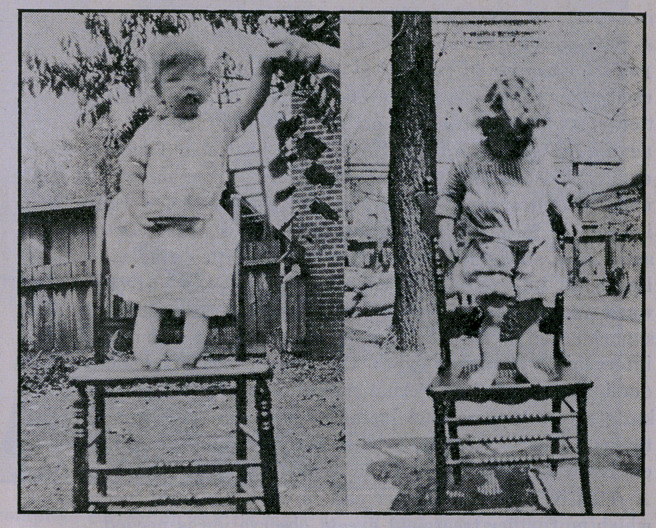


**Figure f4:**
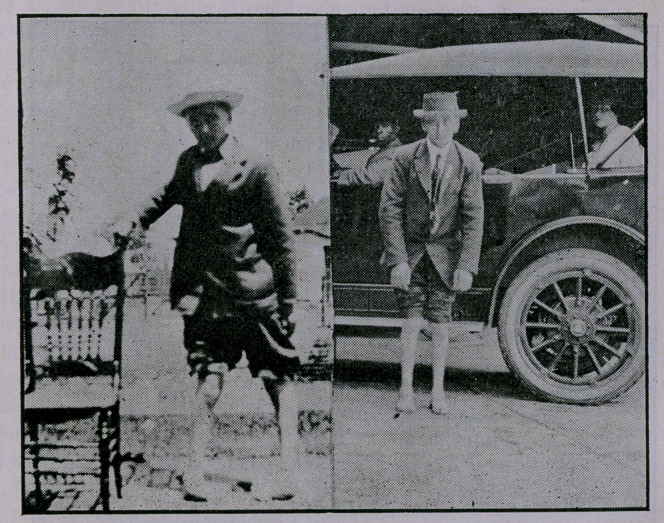


**Figure f5:**
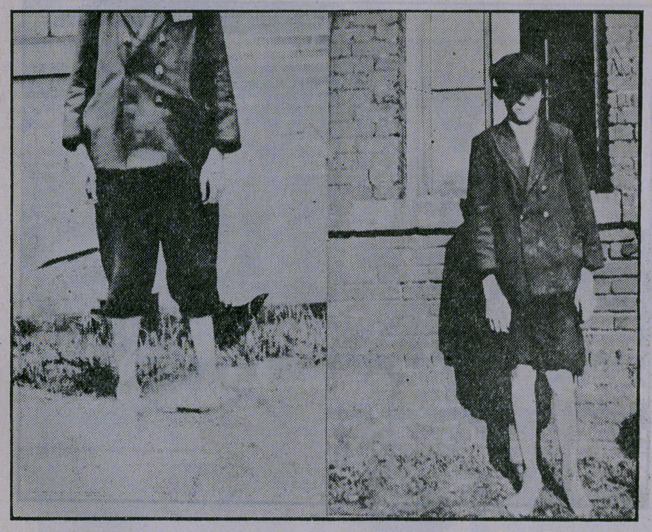


**Figure f6:**